# Supplementary material for: MetaRibo-Seq measures translation in microbiomes
Source: Nat Commun. 2020 Jun 29;11:3268. doi: 10.1038/s41467-020-17081-z (PMC7324362; doi:10.1038/s41467-020-17081-z)
Supplement: Supplementary file 10 — Supplementary Data 7 [file 41467_2020_17081_MOESM10_ESM.zip › File2/Confidence_High_Taxonomy/86985_out.krona.html]

Javascript must be enabled to view this page.

members
magnitude
magnitudeUnassigned
count
unassigned
taxon
rank

86985\_out

7

superkingdom
7
2

phylum
7
1239

class
7
186801

186802
7
order

7
541000
family

7
1263
genus

species
5

SRS015431\_contig\_number\_78195SRS1041164\_contig\_number\_5217SRS104912\_contig\_number\_contig-100\_137.120060SRS1055083\_contig\_number\_9148SRS140492\_contig\_number\_contig-100\_251.195715
2293149

species

SRS097920\_contig\_number\_13732
2293216
1


SRS024663\_contig\_number\_14110
2293177
1
species
